# Linear Associations between Clinically Assessed Upper Motor Neuron Disease and Diffusion Tensor Imaging Metrics in Amyotrophic Lateral Sclerosis

**DOI:** 10.1371/journal.pone.0105753

**Published:** 2014-08-21

**Authors:** John H. Woo, Sumei Wang, Elias R. Melhem, James C. Gee, Andrew Cucchiara, Leo McCluskey, Lauren Elman

**Affiliations:** 1 Department of Radiology, Perelman School of Medicine at the University of Pennsylvania, Philadelphia, Pennsylvania, United States of America; 2 Department of Biostatistics and Epidemiology, Perelman School of Medicine at the University of Pennsylvania, Philadelphia, Pennsylvania, United States of America; 3 Department of Neurology, Perelman School of Medicine at the University of Pennsylvania, Philadelphia, Pennsylvania, United States of America; 4 Philadelphia Veterans Affairs Medical Center, Philadelphia, Pennsylvania, United States of America; 5 Department of Radiology, University of Maryland, Baltimore, Maryland, United States of America; University of Florida, United States of America

## Abstract

**Objective:**

To assess the relationship between clinically assessed Upper Motor Neuron (UMN) disease in Amyotrophic Lateral Sclerosis (ALS) and local diffusion alterations measured in the brain corticospinal tract (CST) by a tractography-driven template-space region-of-interest (ROI) analysis of Diffusion Tensor Imaging (DTI).

**Methods:**

This cross-sectional study included 34 patients with ALS, on whom DTI was performed. Clinical measures were separately obtained including the Penn UMN Score, a summary metric based upon standard clinical methods. After normalizing all DTI data to a population-specific template, tractography was performed to determine a region-of-interest (ROI) outlining the CST, in which average Mean Diffusivity (MD) and Fractional Anisotropy (FA) were estimated. Linear regression analyses were used to investigate associations of DTI metrics (MD, FA) with clinical measures (Penn UMN Score, ALSFRS-R, duration-of-disease), along with age, sex, handedness, and El Escorial category as covariates.

**Results:**

For MD, the regression model was significant (p = 0.02), and the only significant predictors were the Penn UMN Score (p = 0.005) and age (p = 0.03). The FA regression model was also significant (p = 0.02); the only significant predictor was the Penn UMN Score (p = 0.003).

**Conclusions:**

Measured by the template-space ROI method, both MD and FA were linearly associated with the Penn UMN Score, supporting the hypothesis that DTI alterations reflect UMN pathology as assessed by the clinical examination.

## Introduction

Amyotrophic Lateral Sclerosis (ALS) is a progressive neurodegenerative disease of unknown cause, whose diagnosis requires evidence of both Upper Motor Neuron (UMN) and Lower Motor Neuron (LMN) involvement [1]. UMN disease is primarily assessed clinically, by examining muscle tone and spasticity and eliciting abnormal or pathologic reflexes. Many studies have reported the use of Diffusion Tensor Imaging (DTI) to study the UMN in ALS [2]–[12], generally finding local alterations in the diffusion properties of molecular water within the corticospinal tract (CST). These abnormalities are most commonly characterized by elevations in Mean Diffusivity (MD) and reductions in Fractional Anisotropy (FA).

Studies correlating these DTI changes with clinical assessments of disease severity have been inconsistent. Some groups report correlation with clinical UMN metrics and other measures such as the Revised ALS Functional Rating Scale (ALSFRS-R) or duration of disease [2]–[8], but others find poor or no correlation at all [9]–[12]. While some of this inconsistency may result from the wide variety of DTI acquisition, processing, and analysis methods that were used in these studies [2]–[12], the unclear relationship between DTI metrics and clinical measures of UMN dysfunction does call into question exactly what DTI is measuring and how well it may serve as a reliable and useful biomarker of ALS. Therefore, the present study investigated the relationship between clinically assessed UMN disease and mean MD and FA in the CST, using multiple linear regression analyses. Unlike previous work, we used a novel deformable DTI normalization algorithm that enabled definition of a consistent, user-independent region-of-interest (ROI) to estimate mean MD and FA in the CST.

## Materials and Methods

### Ethics Statement

This study protocol was approved by the institutional review board of the University of Pennsylvania. Written informed consent was obtained from all subjects. All clinical investigation was conducted according to the principles expressed in the Declaration of Helsinki.

### Subjects

Thirty-four patients with ALS were recruited for participation from consecutive new patients who presented at the ALS Association Center at Pennsylvania Hospital over a 2-year period, as well as 13 control subjects with no known neurologic disease. Each subject was classified using the Revised El Escorial criteria [1] into one of the following categories: Clinically Definite ALS, Clinically Probable ALS, or Clinically Possible ALS, by one of two experienced neurologists (LM, LE), who also performed all clinical assessments detailed below. Please refer to Tables 1 and 2 for univariate statistics describing the enrolled subject population. Exclusion criteria included: age less than 18 years old, history of other neurological disease, pacemaker or metallic object considered a contraindication to MR scanning, inability to lie flat in the MRI scanner, and vulnerable populations including pregnant women and prisoners. Patients taking riluzole (Rilutek, Bridgewater, NJ) or who were enrolled in other ALS clinical trials were not excluded. Clinical assessments and DTI studies were performed on all subjects enrolled in the study, as detailed below.

**Table 1:** Descriptive statistics of metric demographic and clinical variables.

N = 34 subjects	Range	Mean	SD
Age	34–82 years	55	10
ALSFRS-R score	17–46	36	7
Duration of disease	232–2509 days	811	579
Penn UMN score	2–30	14	8

**Table 2:** Descriptive statistics of categorical demographic and clinical variables.

Category	Subcategory	Result (%)
Sex	Women	16 (47)
	Men	18 (53)
Handedness	Right	28 (82)
	Left	4 (12)
	Unknown	2 (6)
El Escorial Category	Clin. Definite ALS	11 (32)
	Clin. Probable ALS	13 (38)
	Clin. Possible ALS	10 (29)

### Clinical Assessment

At each patient visit to the ALS Center, clinical metrics including the ALSFRS-R score and a summary score of UMN disease burden were recorded. The Penn UMN Score (see Table 3) ranged from 0 to 32, with higher scores corresponding to greater disease burden, and was comprised of components from the bulbar segment (0–4 points), and each of the four limbs (0–7 points for each limb). Reflexes were judged hyperactive if either pathologically brisk, or retained in a weak or wasted limb. For the bulbar evaluation, single points were allotted each for an abnormal jaw-jerk reflex, facial reflex, and palmomental sign. An additional point was added for a score greater than 13 on the CNS-Lability Scale, a measure of pseudobulbar affect [13]. For the upper extremities, single points were allotted each for an abnormal triceps reflex, abnormal biceps reflex, finger flexors, Hoffmann’s sign, and clonus anywhere in the limb. Additional points were added depending upon the Ashworth Spasticity Scale [14], with Ashworth 1 adding 0 points, Ashworth 2–3 adding 1 point, and Ashworth 4–5 adding 2 points. For the lower extremities, single points were allotted each for an abnormal patellar reflex, crossed adduction, abnormal ankle reflex, Babinski’s sign, and clonus, and similar to the upper extremities, assessment of spasticity added an additional 0 to 2 points.

**Table 3:** Penn UMN Score.

Bulbar Subscore (total 0–4):	
0–1	Increased jaw-jerk reflex
0–1	Increased facial reflex
0–1	Present palmomental sign
0–1	Score >13 on CNS-Lability Scale
**Upper Extremity Subscore (0–7)**	
0–1	Increased triceps reflex
0–1	Increased biceps reflex
0–1	Present finger flexors
0–1	Hoffmann’s sign
0–1	Clonus (anywhere in limb)
0–2	Additional points for spasticity:
	Ashworth 1 (normal tone): 0 points
	Ashworth 2–3: 1 point
	Ashworth 4–5: 2 points
**Lower Extremity Subscore (0–7)**	
0–1	Increased patellar reflex
0–1	Crossed adduction
0–1	Increased ankle reflex
0–1	Babinski’s sign
0–1	Clonus (anywhere in limb)
0–2	Additional points for spasticity:
	Ashworth 1 (normal tone): 0 points
	Ashworth 2–3: 1 point
	Ashworth 4–5: 2 points

Important note: To counter the confound of pseudo-normalization of reflexes with advanced LMN disease, raw subscores for each segment (bulbar, limbs) were modified whenever they were found to decrease over time, to the highest of all previous exam visits. For example, if at the current exam visit the right upper extremity UMN score was 2, but previously had been 4, the current score was manually changed to 4.

The sum of modified UMN scores yielded the Penn UMN score, from 0–32.

We then modified these raw scores, depending upon the results of prior exam visits. At any single point in time, the reflex examination may have been confounded in the presence of advanced LMN disease, so that a previously abnormal reflex may have “pseudo-normalized” on later exams [15]. Therefore, we modified the raw score for each segment to the highest of all previous visits, in order to reflect UMN disease more accurately in the presence of concurrent LMN disease. For example, if at the current exam visit, the right upper extremity UMN score was 2, but previously had been 4, the current score was manually changed to 4. The sum of these modified UMN scores thus yielded the Penn UMN Score, from 0 to 32.

### Diffusion Tensor Imaging

At a separate visit, Diffusion Tensor Imaging of the brain was performed on a 3.0T whole-body scanner (Siemens Trio, Erlangen, Germany), using an 12-channel phased-array head coil, and a 30-direction single-shot spin-echo diffusion-weighted echo-planar sequence, GRAPPA acceleration of 3. The diffusion-sampling scheme consisted of four images with no weighting (b = 0 s/mm^2^), followed by measurements along 30 non-collinear/non-coplanar directions isotropically distributed in space (b = 1000 s/mm^2^). Other parameters: TR = 6700 ms, TE = 85 ms, NEX = 3, FOV = 245×245 mm, matrix = 112×112, slice = 2.2 mm, gap 0 mm, voxel size 2.19×2.19×2.2 mm^3^, scan time 13 minutes. The time interval from the clinic visit to the DTI scan date varied from 57 days before to 43 days after the scan. A similar MRI scan was performed for each control subject, using the same scanner.

### DTI Normalization

**Figure 1:**
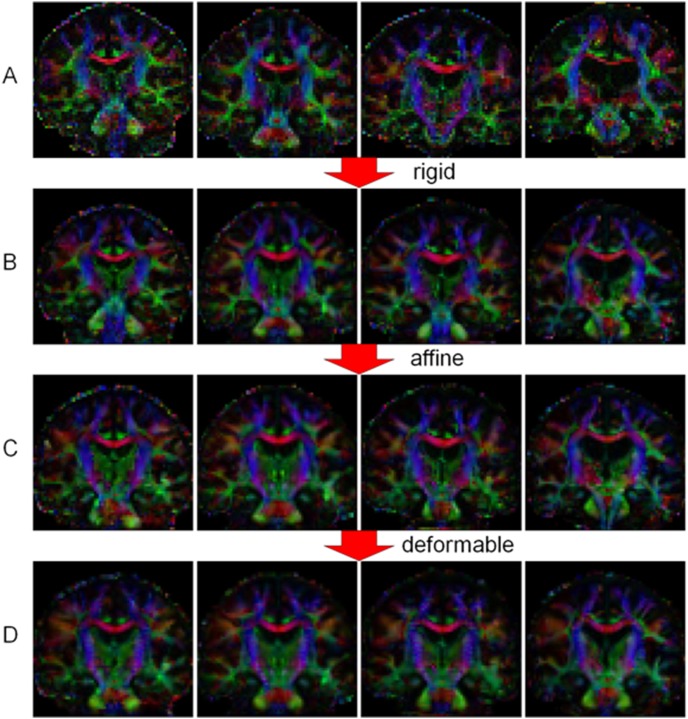
Demonstration of the deformable DTI normalization algorithm used in DTI-TK. Row (A) shows color maps of DTI data (analogous coronal FA maps) of 4 individuals, with variations in head positioning, shape, and volume. Row (B) shows results after the initial registration to the IXI brain DTI template, with improved positioning. Row (C) shows results after applying optimized affine transforms, which only partly correct for shape and volume variations. Row (D) shows results after applying optimized deformable transforms, with considerably improved alignment of white matter structures and correction for shape and volume differences. These spatial transforms enable tractography, ROI definition, and statistics measurement to be performed in template space.

The DTI data on the 34 subjects with ALS were spatially normalized using a high-dimensional deformable registration algorithm that explicitly optimized tensor orientation for alignment of white matter structures, described in more detail elsewhere [16], as implemented in the software DTI-TK (freely available at www.nitrc.org/projects/dtitk) [17]. Image pre-processing steps included correction for motion and eddy-current distortion using standard affine registration methods, and automated brain segmentation to exclude the scalp. Tensors were estimated voxel-by-voxel using standard least-square fitting methods, and all non-positive-definite tensors were zeroed. An initial bootstrap template was generated by affine transforms registering the tensor data to a freely available DTI template based upon the IXI brain database [18], and the transformed data were averaged voxel-by-voxel. This initial template was then improved by an iterative process, at each step first registering the tensors to the template, and then forming a new template by averaging the registered tensors. The process continued until the change between templates from consecutive iterations became sufficiently small, using the squared Euclidean Distance as the tensor metric. This algorithm was applied first with affine, then with deformable (hierarchical dense piecewise affine) spatial transforms, to yield a final population-specific tensor template. The transformed DTI data were assessed subjectively for alignment, and misaligned data were discarded from the analysis. Tensor metrics of FA and MD were calculated voxel-by-voxel using standard formulas of the eigenvalues of the transformed tensors in template-space. The investigator performing these post-processing steps was blinded to the clinical data. Figure 1 demonstrates how the algorithm was able to align DTI data. In a separate step, the DTI data from the 13 controls were individually normalized to the 34-subject template, also using the DTI_TK software.

### Tractography

**Figure 2:**
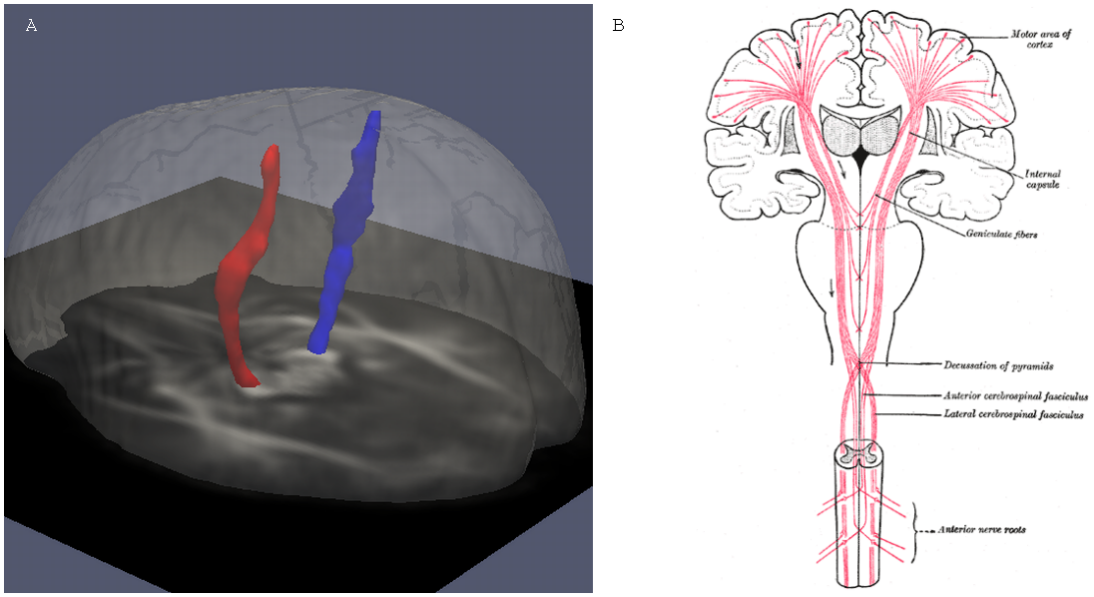
ROIs corresponding to the right and left CST, used for template-space measurements. Figure A depicts the regions-of-interest (ROIs) corresponding to the right (blue) and left (red) corticospinal tracts in template-space, as defined using deterministic tractography. These ROIs were used to measure Mean Diffusivity (MD) and Fractional Anisotropy (FA) in all subjects. As a reference, figure B shows the course of the corticospinal tracts through the brain, taken from http://commons.wikimedia.org/wiki/File:Gray764.png.

Deterministic fiber tractography was performed in the tensor template using the standard FACT method (Fiber Assignment by Continuous Tracking) developed by Mori et al. [19], and implemented in MRIStudio software (freely available at www.mristudio.org). The CST was determined by the following procedure, as outlined by Wakana [20]. Tracking parameters included an FA threshold of 0.20 and an inner product threshold of 0.75 (41°). First, an ROI was drawn by the investigator around either cerebral peduncle, at the axial level showing the superior cerebellar peduncle decussation – identified on color maps by its transverse orientation – and all streamlines passing through this first ROI were displayed. These streamlines were followed superiorly, until the branching point separating the primary motor pathway from all other pathways (i.e. those that do not proceed to the precentral gyrus, as identified based upon anatomic landmarks), and a second ROI was placed at an axial level just above this branching point. The “AND” operation of MRIStudio identified all streamlines passing through both ROIs. The resulting fiber tract data were exported as voxelized binary masks, which served as template-space regions-of-interest in which mean FA and mean MD could be estimated (see Figure 2). To combine the left- and right- side data, the following weighted-average strategy was used: data from each side were first standardized to a mean of 0 and a standard deviation of 1 (for the sample population of 34 subjects), and then averaged together to yield a single bilateral measure for both FA and MD.

A similar “control tract” analysis was performed to obtain DTI measures in the forceps major, the fibers connecting the occipital lobes via the splenium of the corpus callosum, again using the method as outlined by Wakana [20]. Two ROIs were drawn encompassing the entire left and right occipital lobes, in the coronal plane selected at the posterior edge of the parieto-occipital sulcus, as determined on a parasagittal plane at the level of the cingulum. Again, the “AND” operation of MRIStudio identified all streamlines passing through both ROIs.

### Statistics

All tests of significance were two-sided with type 1 alpha error of 0.05, unless otherwise indicated. The Mann-Whitney-U statistic was tested to determine if MD or FA was significantly different between groups of ALS patients and controls, in the CST. Multiple linear regression models (“full models”) were applied separately using either MD or FA, in the CST or in the forceps major, as the dependent variable, and using the Penn UMN Score, ALSFRS-R score, and duration of disease as independent variables, along with El Escorial category, age, sex, and handedness as covariates. Indicator (dummy) variables were used for the categorical data (i.e. El Escorial category, sex, and handedness). Overall significance of a regression model was determined by a standard analysis-of-covariance. Separate analyses (“limited models”) were also performed excluding the Clinically Possible ALS patients, using either MD or FA in the CST as the dependent variable, and their respective significant predictor variables determined from the full model. All statistical analyses were performed using STATA 12.1 (College Station, TX: StataCorp LP).

## Results

There was a significant difference in both MD (Mann-Whitney-U, Z = −2.97, P = 0.003) and FA (Mann-Whitney-U, Z = 2.52, P = 0.01) measured in the CST, between ALS patients and controls.

**Figure 3:**
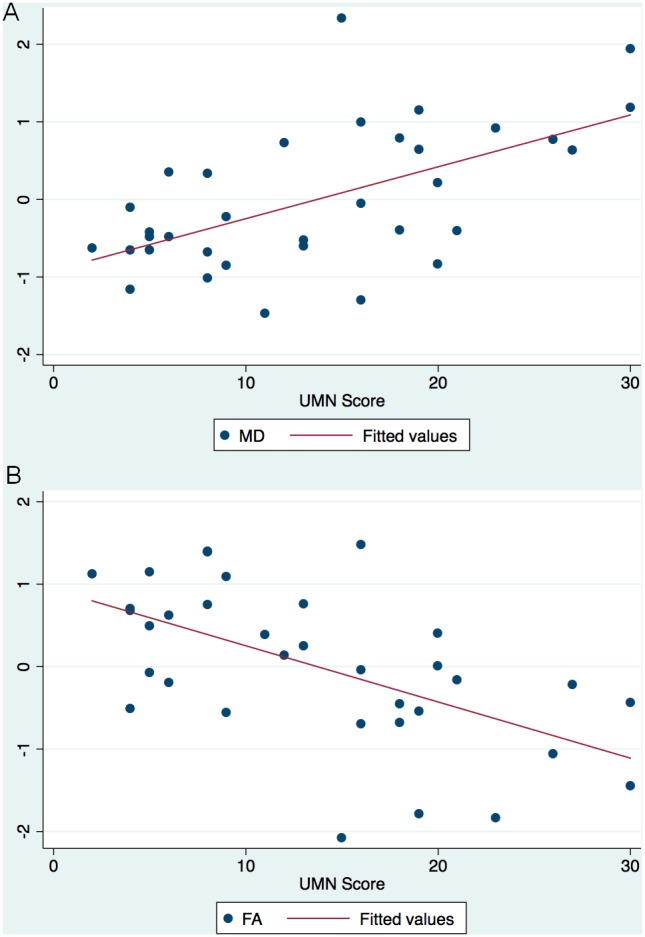
Scatterplots of DTI metrics and the Penn UMN score. (A): The scatter matrix of MD (unitless) vs. the Penn UMN Score (unitless from 0–32) shows an approximate linear relationship. Superimposed simple regression line is also shown. (B): The scatter matrix of FA (unitless) vs. the Penn UMN Score (unitless from 0–32) shows an approximate linear relationship. Superimposed simple regression line is also shown.

The scatterplots of the DTI metrics (MD and FA) measured in the CST and the clinical metrics (Penn UMN Score, ALSFRS-R score, and duration of disease) are summarized in a scatterplot matrix (see Figure S1). Approximate linear relations were suggested between the DTI metrics and the Penn UMN Score as well as the ALSFRS-R score. No significant association was suggested between the DTI metrics and duration of disease. In figure 3, scatterplots of the DTI metrics MD (3A) and FA (3B) measured in the CST are shown vs. the Penn UMN score with superimposed simple regression lines.

**Figure 4:**
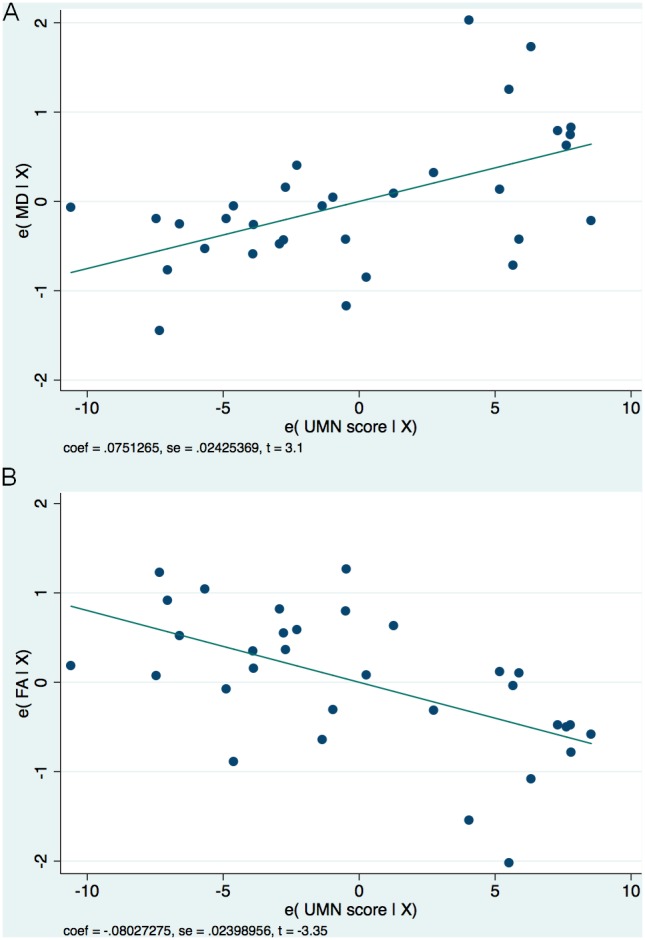
Added variable plots showing the effect of adding the Penn UMN score to the regression models. (A): Added variable plot upon adding the Penn UMN Score to the MD regression model. The Penn UMN Score is a significant predictor in the model, with coeff = 0.075, t = 3.1, P = 0.005. (B): Added variable plot upon adding the Penn UMN Score to the FA regression model. The Penn UMN Score is a significant predictor in the model, with coeff = –0.08, t = –3.35, P = 0.003.

Please see tables 4 and 5 for the analysis-of-covariance tables listing the significance of the predictors in the regression models. For MD in the CST, the full regression model was significant (p = 0.02). The Penn UMN Score was a significant predictor (p = 0.005), as was age (p = 0.03). The ALSFRS-R score and duration-of-disease were not significant predictors (p = 0.48, 0.74, respectively), nor were handedness (p = 0.79) or sex (p = 0.53). Post-estimation analysis showed no significance among the 3 El Escorial categories (p = 0.74). Please see figure 4A showing the added-variable plot (partial regression plot) showing the effect of adding the Penn UMN score to the MD regression model. The limited regression model excluding the Clinically Possible ALS patients but including only the Penn UMN Score and age as predictors was still significant (p = 0.007). The Penn UMN Score (p = 0.003) remained a significant predictor, and its coefficient remained within the confidence interval from the full model analysis. However, age was not a significant predictor in this model (p = 0.10).

**Table 4:** Analysis of covariance table for full regression models.

	MD		FA	
Source	df	p-value	Df	p-value
UMN score	1	0.005	1	0.003
ALSFRS-R	1	0.48	1	0.28
Disease duration	1	0.74	1	0.09
Age	1	0.03	1	0.20
Sex	1	0.53	1	0.25
El Escorial category	2	0.74	2	0.99
Handedness	1	0.79	1	0.22

**Table 5:** Analysis of covariance table for limited regression models, excluding Clinically Possible ALS.

	MD		FA	
Source	df	p-value	df	p-value
UMN score	1	0.003	1	0.01
Age	1	0.10		

For MD in the forceps major, the regression model was not significant (F = 0.72, p = 0.67).

For FA in the CST, the regression model was also significant (p = 0.02). Again, the Penn UMN Score was a significant predictor (p = 0.003). The ALSFRS-R score and duration-of-disease were not significant predictors (p = 28, 0.09, respectively), nor were age (p = 0.20), handedness (p = 0.22), or sex (p = 0.25). Again, post-estimation analysis showed no significance among the 3 El Escorial categories (p = 0.99). Please see figure 4B showing the added-variable plot (partial regression plot) showing the effect of adding the Penn UMN score to the FA regression model. The limited regression model excluding the Clinically Possible ALS patients but including only the Penn UMN score as a predictor was still significant (p = 0.01), and the Penn UMN Score (p = 0.01) remained a significant predictor, with its coefficient within the confidence interval from the full model analysis.

For FA in the forceps major, the regression model was not significant (F = 0.62, p = 0,76).

## Discussion

DTI is a powerful non-invasive method to assess the integrity of white matter tracts of the upper motor neurons in ALS. In their seminal report, Ellis et al. found measurable MD increases and FA decreases in the corticospinal tracts (CST) of patients with ALS [2]. They also reported that FA correlated with clinical measures of UMN involvement, but not with disease duration, whereas MD correlated with disease duration, but not with measures of disease severity or UMN involvement. Several other early groups similarly found FA in the CST to be a potential marker of disability, as measured by ALSFRS-R [3], [4], [5], and some reports also corroborated the potential of MD as a marker of disease duration [4], [5]. Not all reports were consistent, however: one study reported no correlation between either FA or MD and ALSFRS-R [9]; another reported no correlation between DTI metrics (FA or MD) and clinical measures (disease duration, disease severity, or extent of UMN disease) [10]. One potential limitation common to these early studies was their use of user-drawn ROIs to define the CST, which likely contributed significant error to the DTI measures, as well as generally low numbers of subjects, resulting in reduced power.

More recent reports using newer analysis methods have not clarified the relationship between DTI metrics and clinical measures of disease. One study using probabilistic tractography found no association between FA and rate of disease progression [11]. On the other hand, another study found areas in the brain, both in the CST and outside it, with significant correlations between FA or MD measures and ALSFRS-R [6], using both a voxel-based analysis and tract-based spatial statistics (TBSS). Others used deterministic tractography and TBSS to show significant correlations between CST FA and clinical measures of UMN disease, but not with MD, nor with ALSFRS-R [8]. Finally, a very recent report using High-Angular Resolution Diffusion Imaging (HARDI) and probabilistic tractography, despite the better modeling of the diffusion tensor allowed by the HARDI sequence, found no correlation between ALSFRS-R scores and FA in the CST [12]. The wide variety of DTI analysis methods and requisite multivariate statistical tests likely contributed to the variability in these results.

In this study, we found that both MD and FA, when measured throughout the CST using a DTI normalization algorithm, and appropriately controlled for covariates, showed significant linear associations with our clinically derived Penn UMN Score. This finding contrasted with prior reports, which had suggested that FA was a significant marker for disease severity, but not MD. Since both FA and MD were derived from the same set of three eigenvalues that diagonalized the tensor, our finding should not have been surprising. Indeed, a post-hoc analysis showed a high negative correlation between MD and FA (–0.80) in our study data, suggesting a strong interdependence between these two DTI measures. To avoid statistical problems with multiple comparisons, we chose to limit our analysis of DTI metrics to MD and FA, the two most commonly studied.

Our study also found that the clinical metric that best related to DTI changes was the Penn UMN Score, not ALSFRS-R or duration of disease. The UMN score we described, which partially accommodated for a confounded UMN examination in the presence of possible LMN disease by incorporating prior visit data, probably represented an improvement over purely cross-sectional assessments of UMN disease, although it still would not correct for the confounding possibility of LMN degeneration at the initial time point. For ALSFRS-R, the poor contribution to the regression model was not surprising, since this score has been known to be affected by both UMN and LMN disease. The reason why our study failed to corroborate the previously suggested relationship between DTI metrics and duration of disease was not immediately apparent, but may have been due to inherent differences in the patient populations studied. Notably, the duration-of-disease predictor fell just short of significance (p = 0.09) in the full regression model for FA.

The regression analyses also found that El Escorial category of the patient was not a significant contributor to either regression model. Furthermore, the Penn UMN score remained a significant predictor in the regressions even after excluding Clinically Possible ALS cases. These results suggest that all patients with ALS, regardless of the distribution of their clinical manifestations, share the same underlying relationship between UMN disease and DTI changes. Therefore, future research studying the utility of DTI as a biomarker in ALS may include patients in the Clinically Possible ALS category.

Our method to measure the DTI metrics, using a high-dimensional algorithm to normalize the data into a common spatial frame, and template-space tractography to define the CST, had important advantages over methods used in other research articles. First, the method avoided user definition of ROIs, which not only would have been time-consuming, but also could have added error and bias. In contrast, using our method, once the CST was defined by tractography in template-space, subsequent measures for all subjects were performed using exactly the same, consistently defined, unbiased ROI. Indeed, prior studies using more variable ROIs to define the CST may have contained significant measurement error and/or bias, which may have obscured the true relationship between DTI metrics and clinical measures. Finally, our method also would be more suitable for any future large-scale studies, inasmuch as individual tractography or ROI definition need not be performed. All that said, adding a normalization step may have introduced additional pitfalls in the DTI analysis, as pointed out by Jones and Cercigagni [21]. Despite these pitfalls, we found a significant relationship between the DTI metrics and clinical measures.

We purposefully chose a large ROI encompassing both sides of the corticospinal tract to perform DTI measures in this study. Most importantly, this single summary metric of DTI abnormality avoided the multiple-comparison problem that would complicate voxel-based or tract-based methods, and therefore simplified the statistics, albeit at the expense of spatial selectivity. Previous studies have shown variable abnormalities throughout the CST (3, 9). Therefore, we chose the large-ROI method in order to make it the most robust, capturing potential abnormalities throughout the corticospinal tract, while preserving power. As a further benefit, a single summary measure of DTI abnormality also would have more straightforward application as a diagnostic test, or as an endpoint in clinical trials.

It is important to understand that the regression analysis performed in this study can only test for linear associations between the Penn UMN score (a clinical assessment) and DTI metrics in the CST (an MRI measure), but establishing this relationship does not imply causality. That is, while we did find that increasing clinical disease burden (i.e. higher Penn UMN scores) is associated with worsening DTI measures in the CST (higher MD, lower FA), we cannot conclude that worsening disease burden directly results in worsening DTI measures. This limitation is intrinsic to the design of our study.

There were several other limitations to our study. While greater than others, the number of subjects in our study, 34, was not large. If multicenter trials are to be performed evaluating DTI as a biomarker in ALS, the clinical and DTI data should be acquired in a uniform manner in order to be compiled. The success of the Penn UMN Score indicates that a similarly comprehensive upper motor neuron exam should be performed and recorded as the optimal clinical measure for validation. Moreover, putative biomarkers should be specified at the outset so that they can be tested in a formal, prospective fashion. Another limitation to our study was the need to sum together all the components of the clinical exam and the DTI exam across body segments. It may be useful in the future to segment DTI metrics, not only left-vs.-right, but also by body part (i.e. bulbar, cervical, lumbosacral), and then correlate these with the clinical data. Finally, limitations of the diffusion tensor model do not allow full definition of the pyramidal tract by deterministic tractography, due to the “crossing fiber” problem. As a result, only a portion of the true pyramidal tract was in fact interrogated by our methods.

One possible concern with our methods is the complexity added by the DTI normalization step, which theoretically could add error to the measures. However, the benefits of the DTI-TK normalization method likely outweigh this complexity and potential error, and the positive results of our manuscript do indicate that the method successfully allows meaningful DTI measurements that correspond to the clinical examination. Two important differences between the DTI-TK normalization algorithm and other methods are the tensor-based metric used by DTI-TK to drive the normalization, unlike the scalar metric of FA used by standard tract-based spatial statistics (TBSS), and the high number of degrees of freedom in the underlying transform used in DTI-TK, unlike the few degrees used by standard voxel-based morphometry (VBM). Given these differences, it is entire possible, though unproven, that the DTI-TK method significantly improved the registration, and this improvement enabled measurement of DTI metrics that could be related to the clinical examination. Indeed, one recent study that compared several methods [22] found DTI-TK to perform the best among 8 normalization algorithms.

At first glance, the use of the maximum prior reflex scores in calculation of the Penn UMN score may seem problematic; however, this modification to the Penn UMN reflex score was performed for an important reason. It is a known phenomenon that a patient’s reflex examination may artifactually “improve” over time in the presence of increasing lower motor neuron (LMN) degeneration – that is, a reflex that was previously hyperreflexic (signaling UMN degeneration) may appear to normalize, and in cases of end-stage lower motor neuron disease, the reflex will abolish. This “improvement” does not reflect recovery of the UMN’s or decreased disease burden, but rather is an indication that there are not sufficient lower motor neurons remaining to generate a reflex. To correct for this artifact, the total UMN score was calculated from the maximum UMN score per segment during the course of the disease.

In summary, we used an unbiased method to measure DTI abnormalities in the CST of patients with ALS, and found significant linear relationships with a clinically-assessed metric of UMN disease, but not with the ALSFRS-R score or duration of disease, when controlled for covariates including the El Escorial category. Contrasting with other reports, we found that both MD and FA showed significant linear relationships with UMN disease. This finding has important consequences for future studies using DTI as a biomarker of UMN disease burden, as both MD and FA should be studied.

## Supporting Information

Figure S1 **Scatterplot matrix of DTI and clinical metrics.** This figure shows a scatterplot matrix of the DTI metrics (MD, unitless; FA, unitless), and the clinical metrics (Penn UMN Score, unitless scale from 0–32; ALSFRS-R, unitless scale from 0–42; duration-of-disease, in days). Linear associations are suggested between the DTI metrics and the Penn UMN Score, as well as ALSFRS-R score, but not the duration-of-disease. The asterisks (*) denote the significant relationships between MD and the Penn UMN Score (p = 0.005) and between FA and the Penn UMN Score (p = 0.003).
(TIF)
