# Supplementary figures and images for: Linear Associations between Clinically Assessed Upper Motor Neuron Disease and Diffusion Tensor Imaging Metrics in Amyotrophic Lateral Sclerosis
Source: PLoS One. 2014 Aug 21;9(8):e105753. doi: 10.1371/journal.pone.0105753 (PMC4140827; doi:10.1371/journal.pone.0105753)

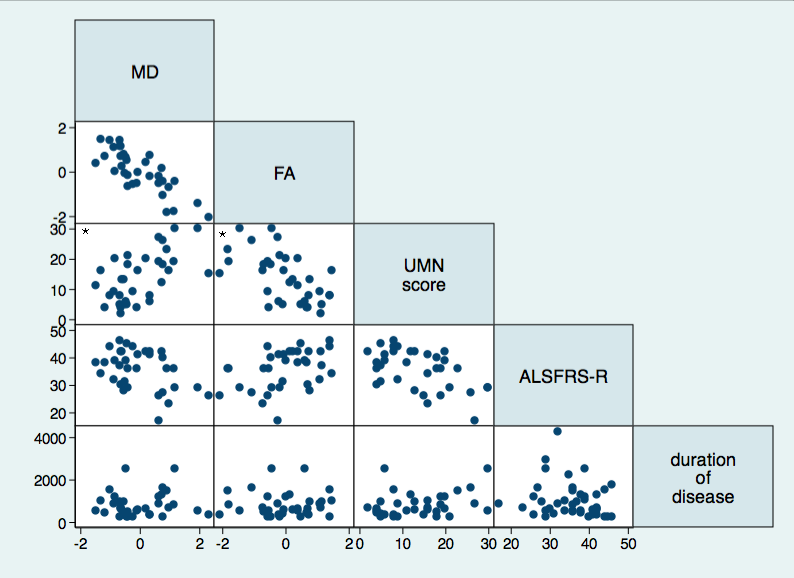

Supplement: Figure S1 — Scatterplot matrix of DTI and clinical metrics. This figure shows a scatterplot matrix of the DTI metrics (MD, unitless; FA, unitless), and the clinical metrics (Penn UMN Score, unitless scale from 0–32; ALSFRS-R, unitless scale from 0–42; duration-of-disease, in days). Linear associations are suggested between the DTI metrics and the Penn UMN Score, as well as ALSFRS-R score, but not the duration-of-disease. The asterisks (*) denote the significant relationships between MD and the Penn UMN Score (p = 0.005) and between FA and the Penn UMN Score (p = 0.003). (TIF) [file pone.0105753.s001.tif]
